# Remembering the opponent: neuronal activation associated with social memory in zebrafish

**DOI:** 10.3389/fvets.2026.1793342

**Published:** 2026-03-20

**Authors:** Luciano Cavallino, María E. Pedreira, Andrea G. Pozzi, María F. Scaia

**Affiliations:** 1 Laboratorio de Neurociencias de la Memoria, Instituto de Fisiología, Biología Molecular y Neurociencias (IFIBYNE), CONICET, FCEyN-UBA, Buenos Aires, Argentina; 2 Instituto de Biodiversidad y Biología Experimental y Aplicada (IBBEA-CONICET), Buenos Aires, Argentina; 3 Laboratorio de Neuroendocrinología del Comportamiento Social, Instituto de Fisiología, Biología Molecular y Neurociencias (IFIBYNE), CONICET, FCEyN-UBA, Buenos Aires, Argentina

**Keywords:** aggression, c-fos, neuronal activation, social memory, zebrafish

## Abstract

Learning and remembering an opponent and the characteristics of a previous encounter may allow individuals to modify their behavior based on that acquired information. In the present study, using a long-term memory paradigm associated with social interactions, we aimed to evaluate the neuronal activation underlying individual recognition and memory of a previous agonistic encounter in male zebrafish. By quantifying the immediate-early gene c-fos, we compared neuronal activation across different telencephalic nuclei of the social decision-making network. Two dorsal nuclei, the medial (Dm) and the lateral (Dl), and two ventral nuclei, the ventral (Vv) and the dorsal (Vd), were evaluated by quantifying c-fos protein by immunohistochemical techniques. Two agonistic encounters between the same pair of opponents were performed. The number of c-fos–immunopositive cells was quantified immediately after the second encounter in fish that either exhibited behavioral evidence of remembering their opponent, behaved as if they did not recognize the opponent due to treatment with an amnesic agent after the first encounter, or belonged to a no-agonistic-encounter group with no physical interaction. We found that the Vv nucleus showed lower activation in individuals who were not exposed to physical interaction than in those who participated in agonistic encounters. The Dl nucleus showed a higher activation only in individuals who would face an unremembered opponent, but not in those who recognized the opponent. Our findings provide a first step toward understanding the neuronal processes underlying opponent recognition and retrieval of prior agonistic experience.

## Introduction

Social experience can modulate behavior and alter interactions between conspecifics (1). Complex animal social behaviors involve a range of cognitive skills, including observational learning and individual recognition. In this sense, long-term memory formation helps improve future responses that are adjusted to past experiences (2). During aggressive interactions, individuals can sense multisensory cues, learn from their encounters, and remember different features of them. Consequently, animals can adjust their behavior to prevent energy waste and injuries, thereby improving animal welfare (3, 4). For this to occur, it is necessary to recognize and remember the opponent and recall this information in subsequent encounters. Social learning has been proposed as a significant factor in the evolution of social behavior (5). Given that cognitive skills must be transmitted and underpinned by neural systems for social systems to develop, it is crucial to characterize neuronal activation and evaluate the brain nuclei involved in this type of memory.

In this regard, an interesting neural substrate to understand how social experience can modulate social behaviors is the Social Decision-Making Network (SDMN), composed of the social behavior network and the mesolimbic reward system (6). While the first circuit regulates social behaviors such as aggression, reproduction, and parenting, the mesolimbic reward system evaluates the salience of perceived stimuli to generate the appropriate behavioral response (6). Even if these circuits have been initially characterized in mammals, in teleost fish a consensus has emerged from neurophysiological, behavioral, anatomical, developmental, molecular, and gene expression evidence suggesting that different areas in the telencephalon and hypothalamus are homologous to specific brain regions in mammals (7). These homologies were supported by substantial neurophysiological and behavioral evidence, including activation and lesion experiments, as well as functional connectivity analyses evaluating how certain regions responded to particular stimuli and the interconnections between different areas and known cell populations (7, 8). Over the last years, zebrafish, *Danio rerio,* have become an increasingly popular model for studying brain development, neural activity, and neuronal circuits related to cognition and emotion in non-human animals (9). Consistent with these findings, multiple studies have investigated the activation of telencephalic nuclei during social interactions in the zebrafish by quantifying various molecular markers indicative of neuronal activation (10–12, 63). In this sense, a well-established technique for evaluating neuronal activation is the quantification of immediate early genes (IEGs). While the expression of these genes is not specific to neurons, in both *in vivo* and neuronal cell cultures, the transcription of early genes was induced by electrical stimulation and by neurotransmitter analog activation (14–16). In this way, the expression of IEGs, including c-fos, occurs rapidly and transiently following synaptic stimulation (17) and neuronal activation, as well as through pharmacological treatment or sensory stimulation (18).

In particular, the relationship between c-fos-expressing neurons and memory has been examined using various experimental approaches. For example, optogenetic experiments suggest that reactivation of hippocampal neurons expressing c-fos during fear conditioning induce mice to exhibit a conditioned response (19), while the inactivation of these neurons causes an impairment in memory retrieval in mice exposed to a context fear learning paradigm (18, 64). In addition to mammals, c-fos quantification has been widely used as a marker of neuronal activity across various species, including zebrafish (20–22). Such is the case of immunohistochemical studies suggesting that exposure of zebrafish to caffeine correlates with an increase in the number of c-fos immunopositive cells across various brain regions (23). Regarding temporal patterns, immunohistochemical studies in rat neocortex suggest that c-fos protein expression peaks at 90 min after the stimulus, with a range of 30 min to 2 h (24). Interestingly, evidence in zebrafish suggests peaks in protein expression at 60 min, although changes in expression can also be observed at 30 min (23, 25).

Learning processes and behavioral modifications can persist through long-term memory formation, which requires synaptic plasticity changes dependent on molecular signaling cascades. These changes can strengthen specific synaptic connections and discrete brain networks (26). In particular, ionotropic glutamate receptors, including N- methyl-D-aspartate receptors (NMDA receptors), play a crucial role in synaptic plasticity. The synapse assembly and disassembly resulting from neuronal activation, understood as an increased expression of its genes, plays a fundamental role in learning and memory (27). In this way, specific brain areas can be linked to particular types of learning and memory by evaluating neuronal activation, neural circuit strengthening, and the effects of inhibiting or activating specific brain regions.

The present study aims to evaluate brain activity associated with the presentation of a conspecific, examining how the retrieval of a memory of a specific individual and/or the characteristics of a previous encounter may modulate neural activity in different nuclei from the SDMN, in particular dorsal and ventral telencephalic areas with reported homologies to specific brain regions in mammals associated with social behavior, learning and memory.

Successive encounters between the same individuals have been widely shown across species to lead to conflict resolution with lower levels of aggression, which may reduce energetic costs and the risk of injury (1, 28–32). In line with this, our previous studies in this species showed that successive encounters between the same opponents were resolved with lower levels of aggression when the second encounter occurred 24, 48, or 72 h after one. In contrast, when the opponent changed and the second encounter involved unfamiliar opponents, aggression levels did not decrease. Moreover, treatment with an amnesic agent (MK-801, an NMDA receptor antagonist) immediately after the first encounter —presumably preventing the formation of memory related to the characteristics of that interaction — also impaired the reduction of aggression in the subsequent encounter, with opponents behaving as if they were facing a novel opponent. Interestingly, this maintenance of aggression levels was observed when the second encounter was against an unfamiliar opponent (28, 29).

Our previous work suggested that individuals that were introduced to a beaker with no pharmacological treatment after the first encounter formed a memory of their opponent and the agonistic encounter. Therefore, when facing the same opponent again, they adjusted their behavior based on previous experience, and the second encounter showed lower levels of aggression; introducing them into a beaker containing the amnesic agent after the first encounter did not decrease their levels of aggressiveness in the second fight. Instead, they behaved as if facing a novel opponent, showing they would not remember their specific opponent (29). Thus, considering these behavioral results, in this study, we will compare brain activity in the group remembering their opponent during the second encounter (e.g., those individuals treated with water after the first encounter) and in the group facing an unrecognized opponent during the second encounter (e.g., those individuals treated with the amnesic agent MK-801 instead of water). Here, and further, brain samples from the Water and MK-801-treated groups in (29) were processed for brain activation analysis to assess the correlates of these behaviors in the Opponent remembered and Opponent not remembered groups.

Additionally, we included a No Agonistic Encounter group consisting of individuals who had no physical or visual interaction. On this basis, by determining neural activity immediately after the second encounter and comparing groups that show a reduction in aggression with those that do not, we can assess the differential effects of remembering, or failing to remember, the opponent and/or the characteristics of the previous encounter. Altogether, these analyses indicate differential activation of specific telencephalic nuclei as a function of social experience and the successful retrieval—or failure to retrieve—long-term memory of previous agonistic encounters.

## Material and method

### Ethics statement

The procedures used in this study followed the institutional guidelines for the use of animals in experimentation (Comisión Institucional para el Cuidado y Uso de Animales De Laboratorio, Facultad de Ciencias Exactas y Naturales, Universidad de Buenos Aires, Protocol 75b/2021) and were under the National regulations (Comité Nacional de Ética En la Ciencia y la Tecnología). All procedures complied with the ARRIVE guidelines and the Guide for Care and Use of Laboratory Animals (eighth ed. 2011, National Academy Press, Washington, p. 220). The euthanasia method was chosen in accordance with the guidelines of the (65). According to AVMA guidelines, individuals were euthanized by rapid chilling followed by decapitation. The animals’ maintenance was carried out with attention to their nutrition, water conditions, and environment to ensure their well-being, and their handling was performed with all necessary precautions to minimize stress. In this sense, the water system was monitored for nitrites, nitrates and amonia, and to maintain conductivity and pH. Animals were maintained in an enriched environment with plants, gravel and shelters, and their mobility and motivation to eat and socially interact with conspecifics were daily monitored.

### Animal and maintenance

Adult zebrafish males, *Danio rerio* (66) (*n* = 21, weight = 0.18 ± 0.01 g, standard length = 2.40 ± 0.03 cm; mean ± SEM) were obtained from commercial aquaria in Buenos Aires, Argentina. One month before the experiments, individuals were acclimated in 20-L community tanks (density = 1 individual/L; 4 different aquariums): 25–26 °C, pH = 7.5/7.8, and a photoperiod of 14 h light: 10 h dark cycle (33). They were fed twice daily with commercial food (Tetra®). For the present study, we analyzed brain samples from a subset of the experiments described in (29) (here these experimental groups are referred to as Opponent Remembered and Opponent not Remembered groups), and we also included a No Agonistic Encounter group.

### Experimental treatments

**Figure 1:**
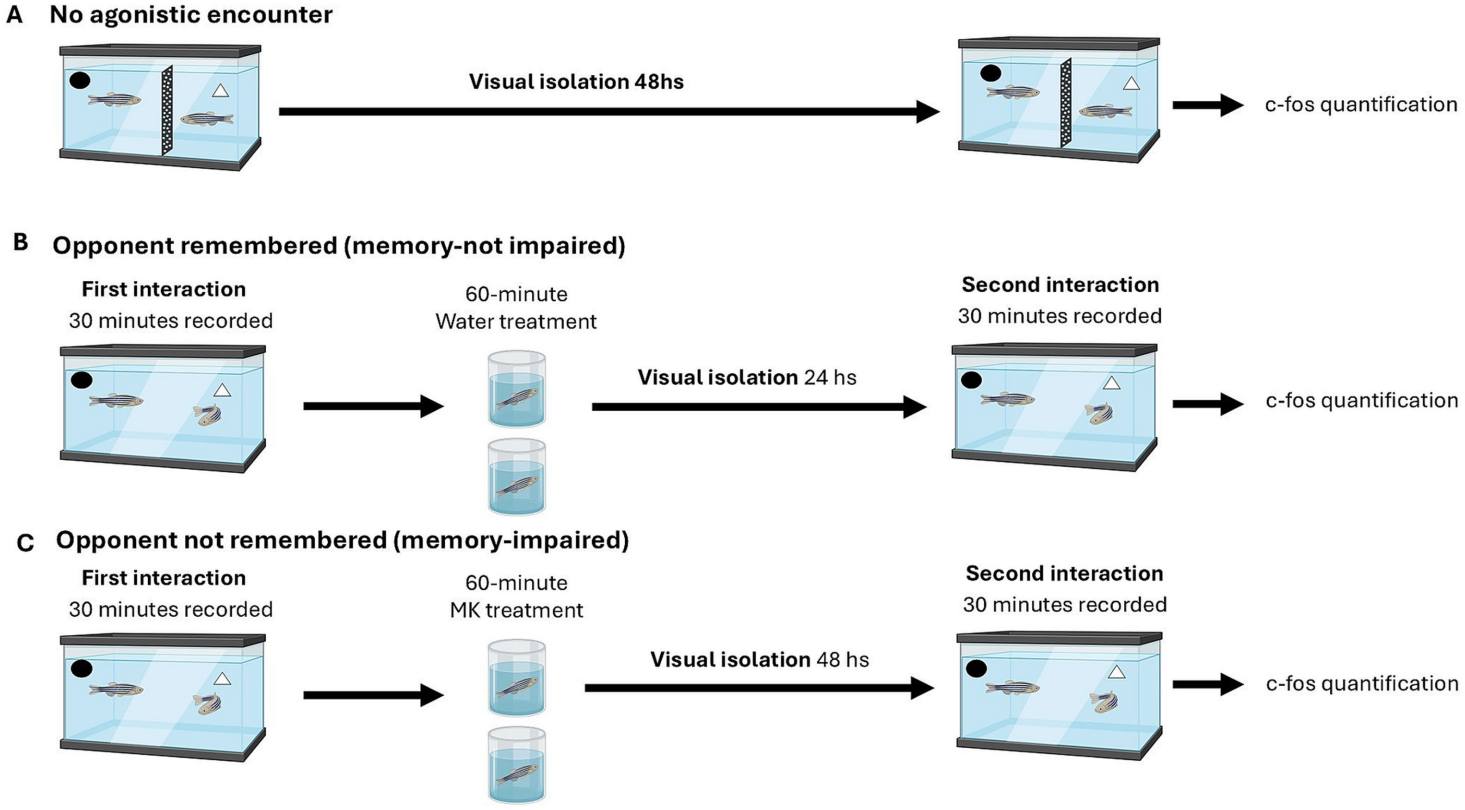
Experimental device and treatments. **(A)** Pairs of zebrafish males were isolated in a 2 L experimental aquarium, separated by an opaque barrier. Forty-eight hours later, individuals were euthanized by cold shock and decapitation, and samples were processed for c-fos quantification. **(B)** Experimental device in which pairs of zebrafish males were exposed to two subsequent agonistic encounters with the same opponent. Fish were allowed to interact for 30 min, and immediately after the first encounter, pairs received a water treatment in a treatment tank, then were returned to the experimental aquarium and isolated for 24 h until they interacted again for 30 min. **(C)** Experimental device in which pairs of zebrafish males were exposed to two subsequent agonistic encounters with the same opponent, but memory was impaired. Fish were allowed to interact for 30 min, and immediately after the first encounter, animals received MK-801 treatment dissolved in water in a treatment tank. Then, fish were returned to the experimental aquarium and isolated for 48 h until they interacted again for 30 min.

To assess the neural substrate related to memory retrieval of previous agonistic behavior, in the present study we analyzed individuals from three experimental treatments: an isolated group without visual or physical interaction, a group that exhibited reduced aggression during the second encounter (exposed only to water after the first encounter), and a group that did not reduce aggression during the second encounter (treated with MK-801 immediately after the first encounter) (Figure 1). After acclimation in community tanks, pairs of size-matched adult male zebrafish from different tanks were placed in a small experimental aquarium (2 L; 15 cm high × 24 cm long × 9 cm wide). The individuals were separated by an opaque perforated barrier that allowed chemical communication but prevented visual and physical contact. This barrier could be removed to allow fighting in the Opponent Remembered (OR) and Opponent Not Remembered (ONR) groups. Water or MK-801 exposure was performed individually in a treatment tank (100 mL; 6 cm high × 10 cm long × 10 cm wide) (29). Same tanks were used for the No Agonistic Encounter group.

No Agonistic Encounter (NAE; *n* = 6): A pair of adult male zebrafish of similar size was placed in the experimental tank, separated by an opaque barrier that allowed chemical but not visual or physical interaction for 48 h. They were then euthanized, immediately fixing heads as described in the following section.

Opponent Remembered (memory-not impaired, OR; *n* = 6): This group consisted of individuals who showed lower aggression during the second encounter than during the first. After an initial encounter resolved at a given level of aggression, individuals were transferred to a treatment tank containing pure water for 1 h and then returned to their experimental tank. Twenty-four hours later, the same individuals were re-paired and engaged in a second encounter, which was resolved with reduced levels of aggression. Immediately after the 30-min encounter, fish were euthanized and their heads fixed as described below. These individuals represent a subset of those behaviorally analyzed in Cavallino et al. (29).

Opponent not Remembered (memory-impaired, OnR; *n* = 9): This group consisted of individuals who did not show a reduction in aggression during the second encounter compared with the first. After the initial encounter, resolved with a given level of aggression, individuals were exposed to MK-801 in a treatment tank for one hour and then returned to their experimental tank. 48 h later, the same individuals were re-paired and engaged in a second encounter, which was resolved with similar or higher levels of aggression than the first encounter. Immediately after the 30-min encounter, fish were euthanized and their heads fixed as described below. These individuals also represent a subset of those behaviorally analyzed in Cavallino et al. (29).

The detailed protocol for the encounters is described in Cavallino et al. (29). Briefly, we assessed whether aggression decreased during the second encounter and measured brain activity immediately after this encounter in these three groups. In all cases, individuals were euthanized by cold shock and decapitation, and samples were processed for c-fos quantification.

### MK-801 treatment

As mentioned, the brains of individuals treated with the amnesic agent are a subset of data from Cavallino et al. (29). Since an evident amnesic effect was observed after 60 min but not after 15, 30, or 45 min, we used a treatment duration of 60 min in this study. We used a concentration of 20 μM based on previous literature (34, 67) and pilot experiments, in which lower concentrations did not produce an amnesic effect. It is important to note that while MK-801 has been reported to have short-term behavioral effects (35, 36), we analyzed behavior and brain activity 48 h after treatment, at which point these residual effects were no longer observed. In particular, we registered reduced activity at 24 h, followed by a recovery of normal activity and behavior at 48 h (29). Thus, we detected changes in the dynamics of the second encounter, depending on whether the opponents remembered each other, rather than the drug’s immediate behavioral effect.

### Behavioral analysis

In summary, the behavioral results reported in Cavallino et al. (28, 29) analyzed the total time engaged in aggressive displays and the number of bites during the period between the first aggressive display and the resolution of the conflict. In all experiments, the first and second encounters showed clear resolutions, allowing comparisons of whether the resolution involved higher or lower levels of aggression. It is also worth noting that we previously ruled out habituation or fatigue as explanations for the observed effect by observing that aggression levels did not decrease in the second encounter when the opponent was new (28, 29).

### c-fos immunohistochemistry

Immediately after the final encounter, individuals were euthanized by a rapid chilling followed by decapitation (American Veterinary Medical Association, AVMA, Guidelines for the Euthanasia of Animals, 2020). Histological processing of the brains was carried out according to the protocol of Scaia et al. (12). Briefly, heads were fixed in 10% formalin in PBS (posphate-buffered saline) for three days. They were rinsed twice in PBS for 30 min each and decalcified in EDTA (ethylenediaminetetraacetic acid) for two days. After two rinses in distilled water, samples were dehydrated in increasing concentrations of alcohol (70, 96, 100%), alcohol/xylene, xylene, and then two periods of 1:30 h in paraffin I and II. Once embedded, they were mounted in paraffin blocks for histological processing. Coronal sections were cut at 7-micrometers using a microtome (Microm HM 350) and gelatin-coated slides. Sections were deparaffinized, hydrated, and rinsed before immunohistochemistry.

c-fos quantification was performed in brain areas involved in the SDMN and with reported homologies to specific brain regions in mammals (6). Regarding ventral telencephalic areas, while the ventral (Vv) and lateral (Vl) zones are the suggested homologies for the mammalian lateral septum, the dorsal zone (Vd) is homologous to the striatum and partly to the nucleus accumbens. When referring to the dorsal telencephalon, while the medial zone (Dm) in teleosts is the suggested homology for the ventral pallium in mammals (basolateral amygdala), the posterior zone (Dp) is the homologous of the lateral pallium, and the lateral zone (Dl) of the medial pallium (hippocampus). As a consequence, brain activity was assessed by c-fos protein expression in the specific areas Vv, Vd, Dm, and Dl, given their relevance to social behavior, learning, and memory.

**Figure 2:**
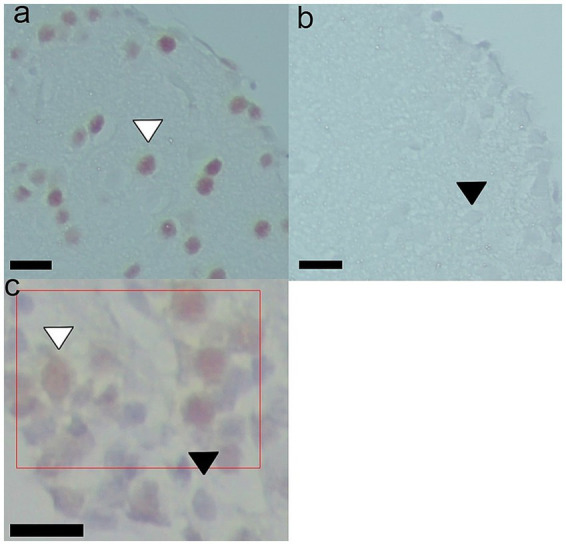
Representative photographs of c-fos immunohistochemistry. A portion of the Dl region is shown for **(A)** positive label for c-fos and **(B)** negative control with no primary antibody incubation. Black arrows indicate unlabeled nuclei, and white arrows indicate positively labeled nuclei. 400 x magnifications. The black bar corresponds to 10 μm. **(C)** The red rectangle represents one of the 1,000 μm^2^ regions in which the amount of c-fos immunopositive cells are quantified (white arrow); unlabeled nuclei can also be observed (black arrow). 400 x magnifications. The black bar corresponds to 10 μm.

The immunohistochemistry protocol began with blocking endogenous peroxidases (3% hydrogen peroxide for 5 min), rinsing, and then blocking nonspecific protein binding sites (5% skim milk for 1 h at room temperature). Slides were then rinsed and incubated at room temperature for 90 min with the primary antibody (polyclonal Goat Anti c-fos Abcam (Ab156802) 1/100 in PBS), followed by overnight incubation at 4 °C. For the negative control, sections were incubated in the same volume of PBS instead of the primary antibody, following the same protocol. The following day, slides were removed from refrigeration and incubated for an additional 1 h at room temperature. After PBS rinsing, sections were incubated for 1 h at room temperature with the secondary biotinylated antibody (Rabbit antigoat DAKO (E0466) 1/200 in PBS). Sections were rinsed and incubated for 1 h at room temperature in Streptavidin HRP (Chemicon (SA202) 1/400 in PBS), rinsed again and developed using DAB (3,3′-Diaminobenzidine, Cell marque 957D-20). The DAB reaction was terminated with distilled water, followed by counterstaining with hematoxylin. Samples were dehydrated and mounted in Canada balsam. After completing this protocol, photographs of the samples were taken with an Axiocam 208 color camera attached to a Zeiss Primo Star microscope for subsequent analysis and quantification (Figure 2).

### Image analysis

**Figure 3:**
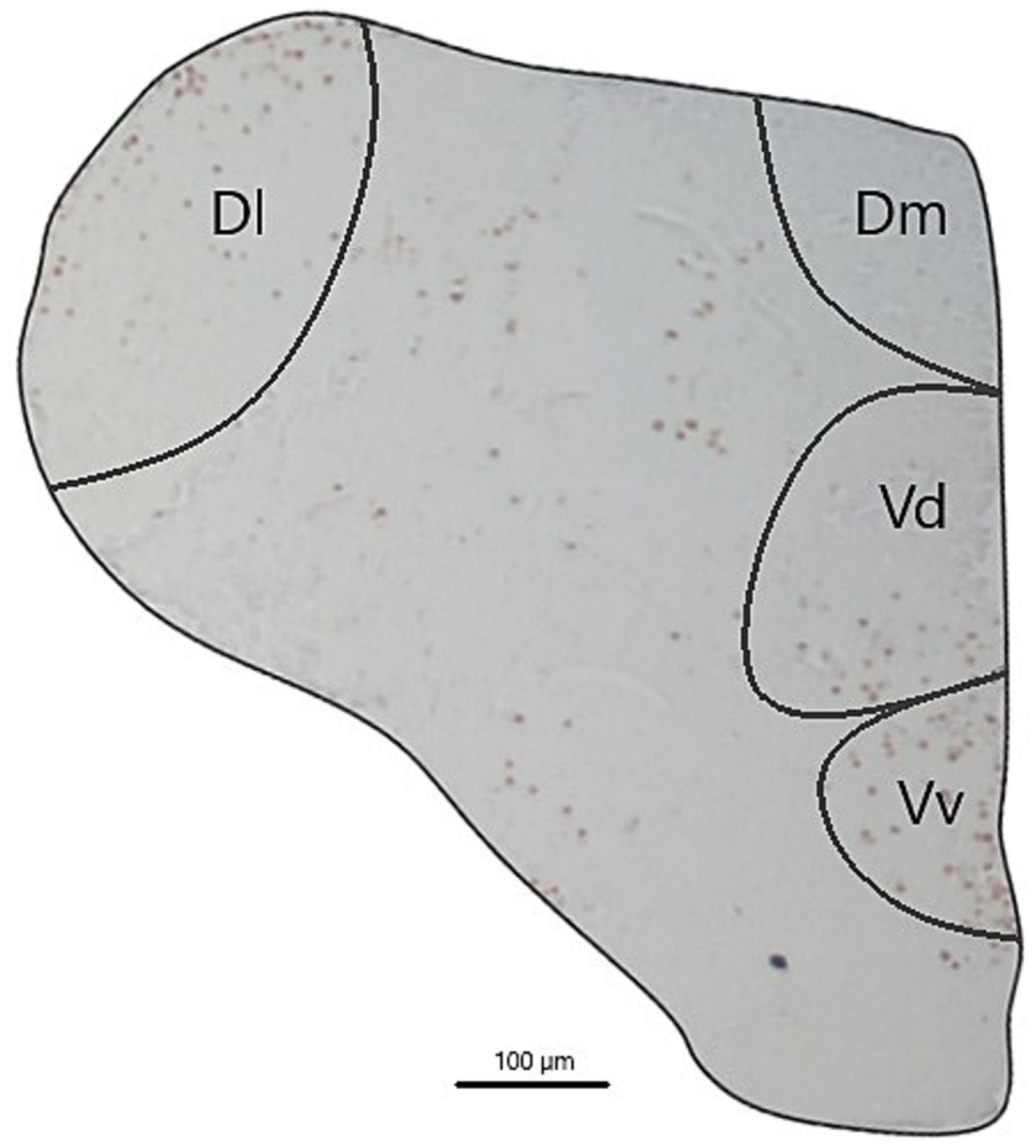
An example of a brain hemisphere analyzed. The regions of interest, Vv, Vd, Dm, and Dl, were determined following the species atlas (37), cross section 85]. Magnification 100X. The black bar corresponds to 100 μm.

Five coronal sections from each region were selected for analysis for each brain, leaving one section between them to avoid multiple quantifications of the same cells. Thus, five cross-sectional slices were quantified for each region per individual. The areas of interest, Vv, Vd, Dm, and Dl, were determined following the species atlas (37). Image analysis and c-fos-positive cell quantification were performed according to the protocol established by Scaia et al. (12). Within each region, two 1,000 μm^2^ rectangles were delineated for each slice, encompassing areas with the highest density of immunopositive cells. The number of immunopositive cells for c-fos within each rectangle was quantified by summing the values obtained across all sections, yielding the number of c-fos cells in a 10,000 μm^2^ area for each brain region. Quantification was performed in a single brain hemisphere, typically the one with the highest number of immunopositive cells. The total area of this hemisphere was quantified to normalize the number of c-fos immunopositive cells per total area of the brain hemisphere for each individual (Figure 3). Image analysis was conducted using Zen 3.3 Blue edition software.

### Data analysis

Since aggression levels did not meet parametric assumptions, data were compared using a non-parametric Wilcoxon rank-sum test (Mann–Whitney U test).

The number of c-fos immunopositive cells per analyzed region (relative to the area of the cerebral hemisphere, IC/TA) was analyzed using linear mixed-effects models with Group and brain region as fixed factors and individual as a random intercept (lmer from the lme4 package and Anova from the car package in R). *Post hoc* pairwise comparisons were performed using estimated marginal means with false discovery rate (FDR) correction for multiple comparisons (emmeans in the emmeans package in R). Results are shown as estimated marginal means ± SE. Model assumptions were assessed by visual inspection of residual and Q–Q plots; residuals showed no substantial deviation from normality.

Complementary, a non-parametric analysis was performed (Kruskal-Wallis test). In those cases in which the Kruskal-Wallis test was significant, a *post hoc* Dunn’s test was performed to compare treatments. Cohen’s d (for balanced comparisons) and Hedges’ g (for unbalanced comparisons) were reported as measures of effect size (38). All median data refer to the number of c-fos-immunopositive cells in a 10,000 μm^2^ region divided by the total area (TA) of each analyzed cerebral hemisphere section (c-fos IC/TA). The data are presented as median and interquartile range (IQR). Statistical significance was set at *p* < 0.05. A non-parametric Wilcoxon paired analysis compared neuronal activation in winners and losers, suggesting no significant differences in MK-treated fish in all four areas (Supplementary Table S1).

A distance-based permutation multivariate analysis of variance [PERMANOVA; (39, 40)] and Principal Component Analysis (PCA) were used to explore individual variability in c-fos-immunopositive cells. PERMANOVA test was performed on Euclidean distance matrices on all four brain areas as variables, with 999 random permutations (41). PCA was conducted to reduce the dimensionality of the dataset, and to explore patterns of individual variability. This approach allowed us to visualize whether the experimental groups formed distinct clusters in a reduced two-dimensional space. Group differences were then formally assessed using PERMANOVA.

A functional connectivity analysis was also conducted to assess correlations between nuclei. Spearman correlations for c-fos immunolabeling between brain areas were calculated for each treatment. Total connectivity (the sum of all the correlations), Strength centrality (the sum of all correlations for each nucleus), and Eigenvector centrality, which considers the connections of each nucleus as well as whether it is connected to other highly connected (i.e., important) nuclei, were calculated using weighted edges with Grafo.eigenvector_centrality (weights = ‘weight’) in R. These correlations and their distributions were compared between treatments using the Kruskal-Wallis test, followed by Dunn’s test, Mann–Whitney U (MWU) test, the Kolmogorov–Smirnov (KS) test, and a permutation test similar to the analysis performed by Scaia et al. (12).

Analyses were done in Python 3.11 and R 4.4.1. Boxplots were performed in GraphPad Prism 6. Scripts and data are available from the corresponding authors upon request.

## Results

### Aggression levels

As previously mentioned, the Opponent Remembered (OR) group (treated with water after the first encounter) showed a decrease in aggression during the second encounter. In contrast, the Opponent not Remembered group (OnR, treated with the amnesic agent after the first encounter) did not show such a decrease (aggression difference between the second and first encounter: Opponent Remembered group: −235.3 ± 62.7 s, *n* = 6; Opponent not Remembered group: 236.7 ± 118.5 s, *n* = 9; W = 54, *p* = 0.0017).

#### Neuronal activation

C-fos immunolabelling in all nuclei across groups were compared. We found a significant interaction between Group and Nucleus (χ^2^(6) = 21.53, *p* = 0.0014). When we compared each nucleus between groups, we found differences only in the Vv and Dl nuclei.

#### Ventral nucleus of the ventral telencephalic area (Vv)

**Figure 4:**
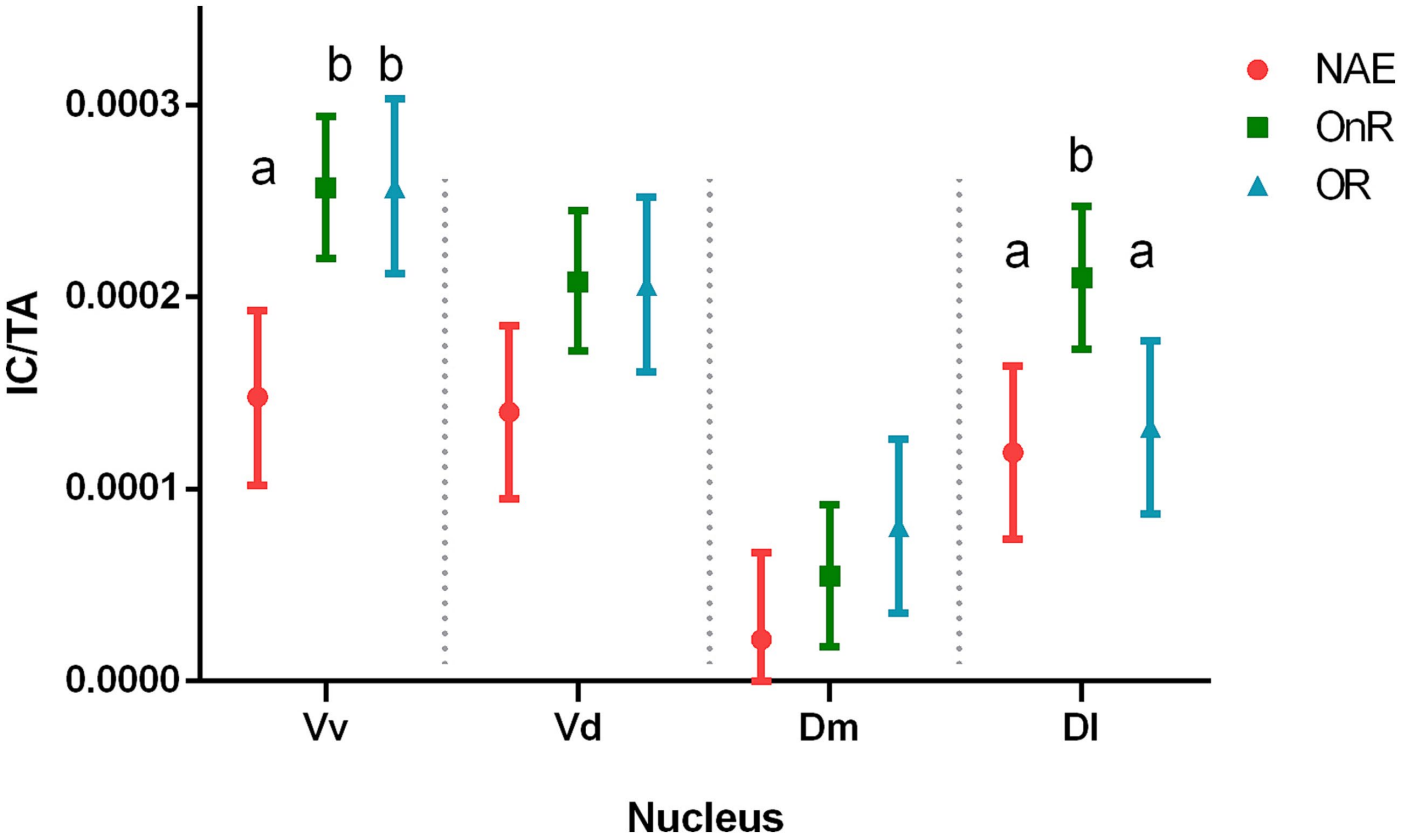
Neuronal activation of each treatment for the analyzed nuclei. Number of c-Fos immunopositive cells/ total area of the cerebral hemisphere (IC/TA) for each nucleus and experimental group. Symbols (red squares, green circles, and blue triangle) represent estimated marginal means derived from a linear mixed-effects model; error bars indicate 95% confidence intervals. Different letters indicate significant differences between experimental groups. NAE, No agonistic encounter; OR, Opponent remembered (memory-not impaired); OnR, Opponent not remembered (memory-impaired); Vv, ventral part of the ventral telencephalon; Vd, dorsal part of the ventral telencephalon; Dm, medial part of the dorsal telencephalon; Dl, lateral part of the dorsal telencephalon.

When comparing the number of c-fos-immunopositive cells per total area among the three groups (No Agonistic Encounter (NAE), Opponent Remembered (OR) and Opponent Not Remembered (OnR)), we found that the NAE group showed reduced immunopositive cells when compared to both the OR group (estimate = −1.10 × 10^−4^ ± 3.19 × 10^−5^ IC/TA, *p* = 0.0016) and the OnR group (estimate = −1.10 × 10^−4^ ± 2.92 × 10^−5^ IC/TA, *p* = 0.0011). No differences were found between the OR and OnR groups (estimate = −2.06 × 10^−7^ ± 2.92 × 10^−5^ IC/TA, *p* = 0.994; Figure 4).

#### Lateral nucleus of the dorsal telencephalic area (Dl)

Individuals not exposed to social encounters in the NAE group showed no differences in neural activation when compared to the OR group (estimate = −1.29 × 10^−5^ ± 3.19 × 10^−5^ IC/TA, *p* = 0.69). Nevertheless, they showed fewer immunopositive cells than the OnR group (estimate = −9.09 × 10^−5^ ± 2.92 × 10^−5^ IC/TA, *p* = 0.0083). Finally, the OR group showed fewer c-fos-immunopositive cells in the Dl than the OnR group (estimate = 7.79 × 10^−5^ ± 2.92 × 10^−5^ IC/TA, *p* = 0.014; Figure 4).

#### Dorsal nucleus of the ventral telencephalic area (Vd)

No significant differences were found between the NAE and OR groups (estimate = −6.63 × 10^−5^ ± 3.19 × 10^−5^ IC/TA, *p* = 0.063), neither when compared with the OnR group (estimate = −6.82 × 10^−5^ ± 2.92 × 10^−5^ IC/TA, p = 0.063) nor between the OR and OnR groups (estimate = 1.96 × 10^−6^ ± 2.92 × 10^−5^ IC/TA, *p* = 0.95; Figure 4).

#### Medial nucleus of the dorsal telencephalic area (Dm)

No significant differences were found between the NAE and OR groups (estimate = −5.86 × 10^−5^ ± 3.19 × 10^−5^ IC/TA, *p* = 0.21), neither when compared with the OnR group (estimate = −3.31 × 10^−5^ ± 2.92 × 10^−5^ IC/TA, *p* = 0.38) nor between the OR and OnR groups (estimate = −2.55 × 10^−5^ ± 2.92 × 10^−5^ IC/TA, p = 0.38; Figure 4).

Complementarily, we performed a nonparametric analysis (Kruskal–Wallis test) and found similar results (Supplementary Table S1). Overall, results reveal increased activation of the Vv nucleus in fish exposed to social encounters, and a significant difference in Dl nucleus activation depending on whether they remembered or did not remember the opponent. It is important to note that, based on the observed behavior, the results suggest that individuals previously treated with water recalled their opponent during the second encounter. In contrast, those previously treated with the amnesic agent faced an opponent they did not remember. Since this represents an initial attempt to identify such differences, we performed the following analysis.

### Individual patterns of neuronal activation across integrated brain nuclei

**Figure 5:**
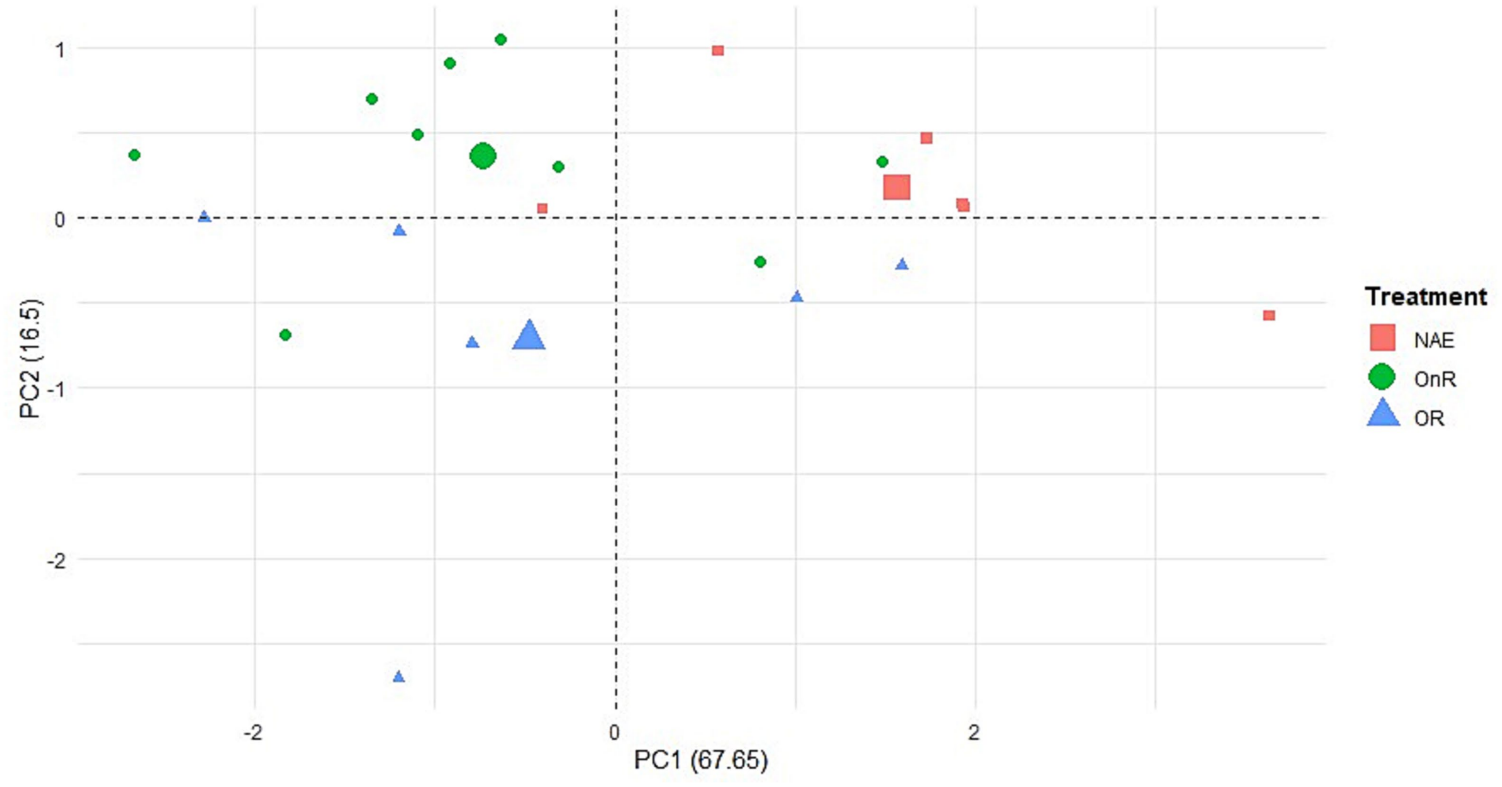
Principal component analysis for the NAE, OnR, and OR group. Red squares indicate individuals’ scores for the NAE group; green circles indicate individuals’ scores for OnR, and blue triangle indicates individuals’ scores for OR group. The largest symbols represent the centroid of each treatment. NAE, No Agonistic Encounter; OR, Opponent Remembered (memory-not impaired); OnR, Opponent not Remembered (memory-impaired).

To explore individual variability in neuronal activation and assess whether the data could be clustered into groups corresponding to the experimental groups, PCA followed by PERMANOVA was performed. The first and second components explained 84.14% of the total variance (PC1: 67.65%; PC2: 16.5%). Table 1 presents component interpretations, showing that areas corresponding to the ventral telencephalon (Vd and Vv) exhibit higher loadings on PC1. In contrast, areas from the dorsal telencephalon (Dm and Dl) contribute more to PC2 (Figure 5; Table 1). When a PERMANOVA was performed, significant differences in PC loadings were found between treatments (*F* = 5.3926, *p* = 0.006, 999 permutations). The NAE group differed from the OnR and OR (NAE vs. OnR1, *p* = 0.024; NAE vs. OR, *p* = 0.039), while no significant differences were found between OnR and OR (*p* = 0.624).

**Table 1:** The proportion of variance indicates the percentage of variance of the nucleus activation explained by each principal component (PC).

Variables	PC1	PC2
Standard deviation	1.6449	0.8123
Proportion of variance	0.6765	0.1650
Cumulative proportion	0.6765	0.8414
Vv	**−0.5392**	−0.0615
Vd	**−0.5551**	0.2240
Dm	−0.4271	**−0.8073**
Dl	−0.4675	**0.5424**

The cumulative proportion shows the explained variance of the PC plus the preceding PC. The loadings are presented for each nucleus (Vv, Vd, Dm, and Dl). The highest loadings for each component are shown in bold. Vv, ventral part of the ventral telencephalon; Vd, dorsal part of the ventral telencephalon; Dm, medial part of the dorsal telencephalon; Dl, lateral part of the dorsal telencephalon.

This analysis, considering all four brain areas as variables, suggests differences in neuronal activation between individuals exposed to physical interaction (OR and OnR groups) and those not exposed to an opponent, which can also be visually separated as a cluster in a principal component analysis (Figure 5). The next step was to evaluate whether functional connectivity among these nuclei differs in the experimental groups.

### Functional connectivity: neuronal correlation

**Figure 6:**
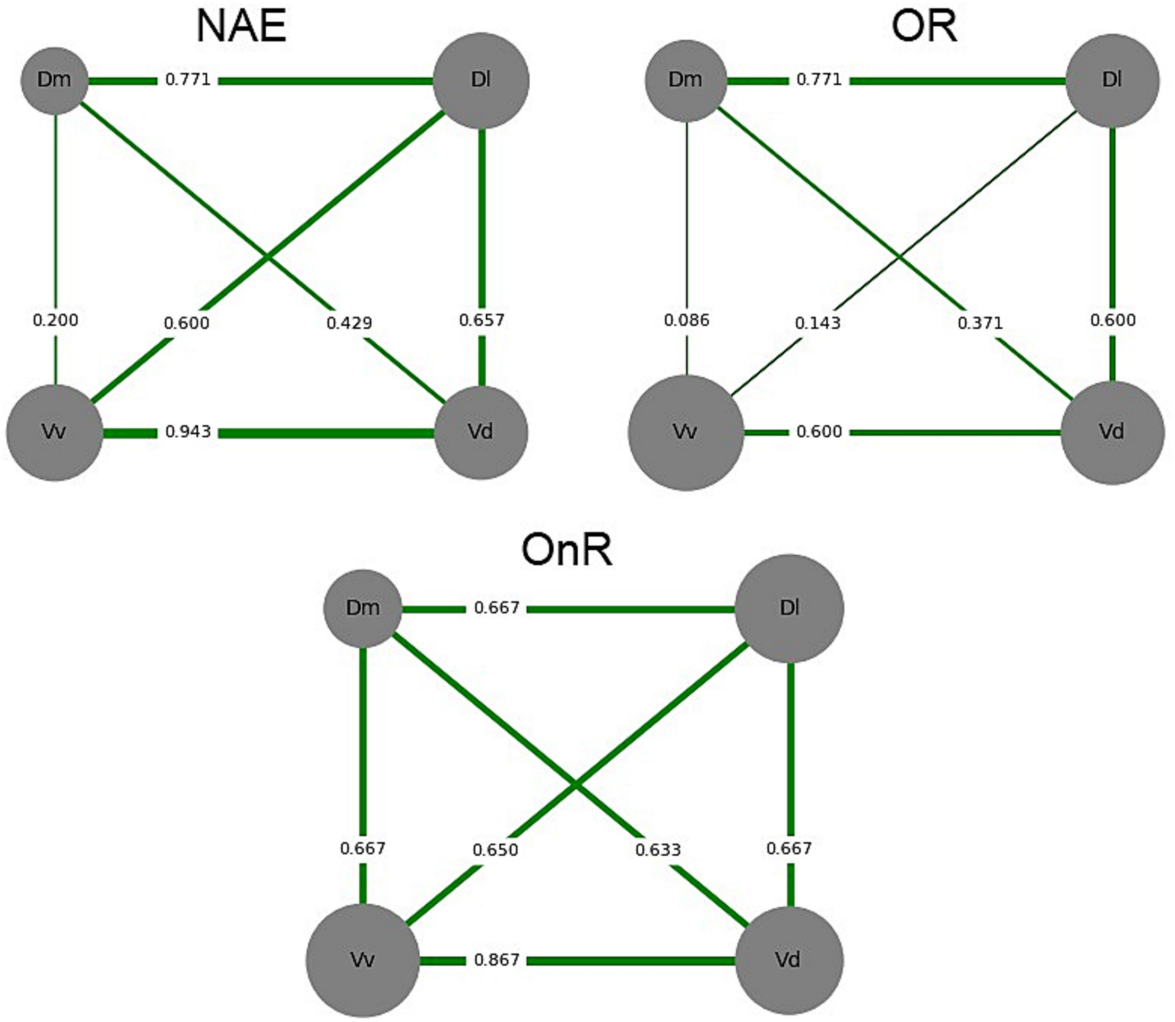
Neuronal correlation of the four nuclei (Vv, Vd, Dm, and Dl) for the three treatments (NAE, OR, and OnR). All the Spearman correlations were positive. The size of each nucleus was proportional to their activation (c-fos IC/TA), and the thickness of the connection lines was proportional to the level of interaction between each pair of nuclei (Spearman’s correlation coefficient). Numbers in each line indicate the Spearman’s correlation coefficient. NAE, No agonistic encounter; OR, Opponent remembered (memory-not impaired); OnR, Opponent not remembered (memory-impaired); Vv, ventral part of the ventral telencephalon; Vd, dorsal part of the ventral telencephalon; Dm, medial part of the dorsal telencephalon; Dl, lateral part of the dorsal telencephalon.

To evaluate the individual activation of each brain nucleus and the relationships among them, correlation networks were constructed for each experimental condition based on correlation matrices of the brain regions analyzed (12, 13). For this, Spearman correlations for the number of c-fos immunopositive cells (c-fos IC/TA) among all the analyzed nuclei (Vv, Vd, Dm, Dl) were calculated for each treatment (Figure 6).

The Spearman correlation matrices for each treatment are shown in Supplementary Table S2. The results suggest that all correlations were positive (i.e., functional co-activation between brain regions), with no negative correlations observed for any pair of nuclei (i.e., functional co-inhibition between brain regions).

Significant differences were found when comparing the OnR and OR groups for both the KS and MWU tests (KS test: statistic = 0.833, *p* = 0.025; MWU test: statistic = 5.0, *p* = 0.0434). No significant differences were found between the NAE and OR (KS test: statistic = 0.333, *p* = 0.930; MWU test: statistic = 10.5, *p* = 0.258) or between the NAE and OnR groups (KS test: statistic = 0.5, *p* = 0.474; MWU test: statistic = 13.0, *p* = 0.468). Similar results were found when Dunn’s test was performed (OnR vs. OR: *p* = 0.0364; OnR vs. NAE: *p* = 0.3997; OR vs. NAE: *p* = 0.2114). Nevertheless, no differences were found when the permutation test was performed (OnR vs. OR: *p* = 0.250; OnR vs. NAE: *p* = 0.762; OR vs. NAE: *p* = 0.368).

### Functional connectivity: total connectivity, strength, and eigenvector centrality

When analyzing total connectivity, although a trend appears to show higher connectivity for the OnR (Total connectivity: 4.15) compared to OR (Total connectivity: 2.57) and NAE (Total connectivity: 3.6), no significant differences were found among the treatments (Supplementary Table S3).

However, when analyzing strength centrality, a significant difference was found between treatments (Kruskal-Wallis chi-squared = 6.038; df = 2, *p*-value = 0.0488, followed by a Dunn’s test). Nuclei in OnR exhibited higher strength centrality than those in the OR group (*p*-value = 0.0142). There were no differences between OnR and NAE (*p*-value = 0.2807) or between OR and NAE (*p*-value = 0.1698). Similar results were found when the permutation test was performed (OnR vs. OR: *p*-value = 0.010; OnR vs. NAE: *p*-value = 0.422; OR vs. NAE: *p*-value = 0.084).

The strength and eigenvector centrality of each nucleus were calculated and ranked from highest to lowest scores according to their importance. The three treatments differed in their rankings of the importance of their nuclei for both strength and eigenvector centrality. When analyzing the first position, the Vd nucleus was most relevant for the OR and NAE groups, and the Vv nucleus was most relevant for the OnR. However, when considering the two most central nuclei in each experimental group, results suggest that while Vd is central for all experimental groups, Dl is highly relevant for NAE and OR, and Vv is only relevant for the OnR group. Additionally, the Dm nucleus was the least relevant for the OnR and NAE treatments, while the Vv nucleus was the least relevant for the OR treatment. The ranking is shown in Supplementary Table S4.

In this regard, connectivity throughout different brain nuclei seems to differ among treatments. Specifically, results suggest significant differences between the OR and OnR in correlation matrices, with a non-significant trend in overall connectivity. Additionally, results suggest a mild difference in strength centrality between experimental groups. Furthermore, even if the centrality of each brain area was not statistically assessed, the order of importance of brain nuclei appears to differ across the three treatments, as assessed by both strength and eigenvector centrality.

## Discussion

The main goal of our work was to evaluate the neuronal activity underlying opponent recognition and long-term memory retrieval associated with social interactions. To disentangle the neural substrates of these processes, we analyzed neuronal activation in brain nuclei associated with aggression, memory, and social interactions using a social memory paradigm by quantifying c-fos expression in male zebrafish. We found higher activation in the Vv nucleus during an agonistic encounter, regardless of whether individuals remembered the opponent, and higher activation in the Dl nucleus in individuals who did not remember the opponent. Furthermore, by using principal component analysis (PCA) and PERMANOVA to assess c-fos expression across all four areas, we were able to differentiate between individuals that participated in an agonistic encounter (Opponent Remembered and Opponent not Remembered groups) and those that did not (NAE group).

It is important to note that the individuals were sacrificed immediately after 30 min had passed following the start of the second encounter (removal of the barrier). In contrast, the NAE group was sacrificed after undergoing similar manipulations but without being exposed to the agonistic encounter. Considering the time required for c-fos expression (24), it is important to emphasize that this method does not quantify neuronal activation corresponding to the resolution of the fight. Instead, this protocol allows the detection of neuronal activation resulting from the recognition and retrieval of a memory of a specific individual and/or the characteristics of a previous encounter, triggered by the interaction during the second encounter (Opponent Remembered group), or neuronal activation related to exposure to a non-recognized opponent (Opponent Not Remembered group). Interestingly, this time frame allows detecting changes associated to the first interactions with an individual who can be recognized and remembered, depending on whether a memory of the previous encounter was formed. In the case of an amnesic effect (previously treated with MK-801) and given the already reported effects on behavioral outcome (29), the neuronal activation observed in this group does not account for recalling the first encounter but rather for a novel interaction between two unfamiliar individuals.

In summary, the individuals had a first encounter, from which they either formed a memory (OR group) or did not (OnR group). Then in the second encounter, they interacted recognizing each other or not, depending on whether they had previously formed a memory. This way, our analysis examined neuronal activation associated with the initial recognition of opponents in the second encounter. As mentioned in the Introduction, our previous work showed that individuals treated with water after the first encounter formed a memory of their opponent, as evidenced by reduced aggression during a second encounter, whereas this memory was impaired when animals were treated with the amnesic agent MK-801 (29). In the present study, in order to assess the neural correlates of these behavioral effects, brain samples from the Water- and MK-801-treated groups reported in Cavallino et al. (29) were used for c-fos expression analysis.

Regarding agonistic encounters, the winner–loser effect has already been described in this and other species, whereby an individual that wins a previous encounter has a higher probability of winning a subsequent encounter against a naïve opponent, and similarly for losers (42). Interestingly, although this effect has been mainly described at short time scales (a few hours), it dissipates within 24 h (43). As a consequence, results using 24-h intervals between encounters suggest that aggression levels remained unchanged when a winner was paired with an unfamiliar loser, whereas aggression decreased between familiar opponents (28). Therefore, these results rule out the possibility that reduced aggression is driven by a winner–loser effect and instead suggest that it is specifically caused by recognition and memory of the opponent. Based on these findings, this evidence suggests that individuals who re-encounter the same opponent and resolve the interaction with reduced aggression recognize and remember the opponent and the characteristics of the previous encounter, thereby adjusting their behavioral strategy accordingly. Conversely, individuals who maintain high levels of aggression during the second encounter (treated with MK-801 after the first one) fail to remember the characteristics of the prior interaction and therefore do not adjust their behavior across successive encounters.

As we mentioned before, c-fos protein levels can be observed between 30 and 120 min after the stimulus, with a maximum peak at 90 min (23–25, 44). In the present work, during the second encounter, individuals were allowed to interact for 30 min after barrier removal, and were sacrificed immediately thereafter. Therefore, the aim of this methodology was not to detect neuronal activation related to fight resolution or aggressive displays *per se*, but rather the impact of the initial phase of interaction during the second encounter. Interestingly, the time window chosen for this methodology thus allows capturing neural activation related to the memory retrieval processes, rather than reflecting activation due to motor activity or stress associated with the second agonistic encounter itself. During these moments, specific nuclei related to social interaction and recalling the memory formed by the previous encounter could be activated in individuals with prior experience against that opponent. However, if the stimulus was perceived as novel (e.g., the Opponent Not Remembered group), we could observe neuronal activation related to the initial stages of evaluating and interacting with a non-remembered opponent. For this reason, we decided to analyze protein expression 30 min after the onset of the first interaction during the second encounter, thereby avoiding confounding effects related to neuronal activation driven by fight resolution and aggressive displays *per se*.

We aim to evaluate brain activity in specific nuclei associated with aggression, emotional learning, and memory. In this sense, a particularly relevant framework for studying the neural correlates of agonistic behavior and social experience is the Social Decision-Making Network, formed by the social behavior network and the mesolimbic reward system [reviewed by (6)]. In mammals, while the social behavior network comprises the preoptic area, the anterior hypothalamus, the ventromedial hypothalamus and the periaqueductal gray, the mesolimbic reward system consists of the striatum, nucleus accumbens, the ventral pallium, the basolateral amygdala, the hippocampus and the ventral tegmental area, being the medial amygdala, lateral septum, and bed nucleus of the stria terminalis (BNST) shared by both networks (6). Given that this network is well conserved across vertebrates, in teleosts different homologies have been suggested, being of particular importance for this study the ventral telencephalic areas (Vv and Vl as homologies for the mammalian lateral septum, and Vd for the striatum and partly nucleus accumbens) and the dorsal telencephalic areas (Dm as homology for the ventral pallium in mammals, Dp for lateral pallium and Dl for medial pallium or hippocampus) (6). Taking this into account, Teles et al. (13) found that, when comparing c-fos mRNA expression for the Dm, Dl, Vv, Vs, and preoptic area (POA) nuclei, zebrafish visually isolated from a conspecific showed lower expression levels in all these nuclei compared to individuals exposed to an agonistic encounter or a mirror interaction (13). These areas are part of the SDMN and represent promising regions for evaluating activation during agonistic encounters. In that study, the authors report the effects of agonistic interactions and the associated aggressive behaviors on brain activation. In our work, the time window chosen allowed us to evaluate activation in the first moments of interaction in the Vv, Vd, Dm, and Dl nuclei.

For the ventral region of the telencephalon, we found that in the ventral nucleus (Vv) those individuals that were not exposed to a social agonistic encounter showed fewer c-fos–immunopositive cells than those that did experience such an interaction, regardless of whether they remembered the opponent or not. Although no significant differences were found in the dorsal nucleus of the ventral area (Vd), a similar trend was observed. This evidence suggests that these nuclei, particularly the Vv, may be involved in social interactions *per se*, regardless of whether the interactions occurs with a familiar or unfamiliar individual. Notably, the Vv was more active during the presentation of a conspecific, independently of whether the opponent was remembered or not, suggesting that this nucleus is related to the agonistic encounter itself and may not be specifically involved in social memory processes within this paradigm.

Interestingly, regarding the Vv nucleus, Fan and colleagues evaluated neuronal activation related to social learning in guppies (*Poecilia reticulata*) by quantifying pS6 (45), a ribosomal protein whose phosphorylation indicates neuronal activation (46). Although pS6 is not an immediate early gene, it has been widely used as a marker of neuronal activation in diverse ethological contexts to study social behavior in fish (10, 12, 47). Fan and colleagues reported increased pS6 immunolabeling in the Vv nucleus during the acquisition of socially mediated threat learning via alarm cues released by conspecifics, highlighting the importance of this nucleus in such processes (45). Similar findings have been reported in zebrafish: Pinho et al. (48) showed that, in a classical conditioning paradigm, increased c-fos expression in the Vv was associated with social learning but not with asocial learning. Together, our results further emphasize the involvement of the Vv nucleus in social learning and corroborate previous evidence supporting its central role in this process.

Regarding the brain areas in the dorsal telencephalon, the medial nucleus (Dm) showed a tendency similar to the Vv, but no differences were found among groups. In contrast, the lateral nucleus (Dl) exhibited more c-fos-immunopositive cells in animals that did not remember the opponent than in those that remembered the opponent or in those without social interaction, with no differences between these two groups. These results suggest that the Dl might be involved in the novel recognition of an individual but not in repeated exposure to the same opponent. We propose that these differences are related to the fact that, following memory impairment, individuals fail to recognize the opponent during the subsequent encounter and behave as if they were facing a novel individual. Accordingly, increased Dl activity may reflect the processing of a socially novel stimulus, indicating a lack of recognition and an associated increase in neural demand. This effect is not observed when the opponent is familiar.

The differences in results between this paper and those presented by Teles et al. (13) can be better understood by considering that in this study we quantified c-fos protein instead of mRNA. It is important to note that mRNA levels do not always correlate with protein levels due to post-transcriptional modifications (23). Additionally, while Teles and colleagues examined c-fos levels in the context of fighting between individuals, this study addresses c-fos expression associated with recognizing a previous opponent during the initial moments of a subsequent interaction, as previously discussed. These findings on neuronal activation, together with our last behavioral results (29), suggest that activation of Dl nucleus can be triggered in response to a novel stimulus (such as the presentation of an unfamiliar individual) and could be involved in the formation of long-term memory related to this novel individual; however, it might not respond in the same way after a repeated presentation.

Nevertheless, it would be necessary to increase the data volume to enhance the robustness of the analysis. It is important to note that the activation and response we refer to is through c-fos expression, as observed in other studies, where not all immediate-early genes respond to the same stimulus. In this sense, Teles and colleagues found differences depending on whether they evaluated c-fos or other immediate-early genes, such as Egr1 (13).

In line with our results, studies in other species report differential activation depending on whether a stimulus is presented for the first time or after several presentations. For example, in the common sparrow, Kimball and colleagues observed that exposure to a series of objects during several days increased c-fos immunolabeling in the caudal hippocampus and the ventral medial arcopallium (homologous to some areas of the mammalian amygdala) only when presented with a novel object the next day, one they had not been exposed to before. However, this increase was not observed if they were presented with an object they had already been exposed to. This suggests that these regions might be involved in recognizing novel objects but would not activate through c-fos with repeated exposure to an object (49). Similar evidence was found in male albino rats, in which individuals without maze exposure showed low levels of c-fos and another IEG, C-jun, as assessed by immunohistochemistry. These expression levels increased considerably when the rats were exposed to a maze for the first time, but the increase was minor with repeated exposure. This effect was observed in various brain regions, including the hippocampus, somatosensory cortex, and cerebellum (50).

It is interesting to note that some studies in fish suggest that the Dl nucleus is mainly associated with spatial learning. In goldfish, active avoidance experiments using conditioning between a green light and an electric shock suggest that lesions in the Dm, but not in the Dl, induced a deficit in testing this conditioning. Conversely, authors observed the opposite in spatial learning experiments: lesions in the Dm did not affect conditioning, but lesions in the Dl did. These results suggest that Dl is involved in spatial learning, while Dm is involved in emotional learning (51). It is important to point out that stress could affect memory and brain activity. Therefore, considering that an unfamiliar stimulus can trigger stress, this factor must be taken into account. Previous evidence suggests that chronic and unpredictable stress reduces spatial memory performance in zebrafish (52), and similar results are reported after acute stress for short-term memory (53). Furthermore, evidence in sea bass suggests a reduction in c-fos expression in the ventral Dl nucleus after non-social stressors, being unpredictable stimulus more stressful than predictable ones (54). In our work, facing a non-remembered opponent could be considered as an unpredictable situation; however, in our case, we observed increased c-fos activity in the Dl nucleus. By contrast, it is important to note that we did not separate the Dl into ventral and dorsal subdivisions. Furthermore, the aforementioned studies evaluated non-social stimuli, whereas we used a social stimulus, namely the presentation of a conspecific. Therefore, this increase in activity could be related to the presentation of a novel social stimulus. Overall, our results showing higher c-fos immunolabeling in the Dl in the OnR group and increased neural activation after exposure to a novel stimulus suggest that the Dl nucleus may be involved in social learning, particularly in the recognition of a novel individual. In contrast, repeated exposure to the same stimulus (OR group) did not elicit the same increase in activation, which may indicate a role of Dl activation in processing socially novel stimuli. However, it will be interesting to confirm the role of Dl in recognizing a novel opponent with other experimental approaches, such as lesions and different ethological contexts.

As previously mentioned, different neuronal markers may provide different types of information. For example, after agonistic encounters in zebrafish neuronal activation in the Dm and Dl nuclei show Teles et al. (13) reported differences in neuronal activation in the Dm and Dl nuclei depending on the marker used (c-fos or egr-1). Similarly, Cerqueira et al. (54) found stress-related differences in the activity of the Dm and Dld nuclei depending on whether c-fos or egr-1 was measured. Furthermore, overexpression of a transcription factor belonging to the Egr family has been shown to enhance object location memory formation in mutant mice (55). Taking this into account, future studies should evaluate additional neuronal markers beyond c-fos in order to better understand the role of each nucleus during social recognition using a broader assessment of neuronal activity. In line with this, it is important to discuss the possibility of a direct effect of MK-801 on c-fos expression. Previous evidence in zebrafish suggests that MK-801 treatment decreases the propagation of spreading depolarization, whereas an increase in extracellular potassium concentration enhances c-fos expression in the tectum (56). In contrast, MK-801 treatment increases c-fos expression in the mouse cortex (57). It is important to emphasize that in both cases these effects were observed immediately or shortly after MK-801 administration, in contrast to the present study in which c-fos expression was measured 48 h after treatment. To our knowledge, there is no evidence of a direct long-term effect of MK-801 on c-fos expression at such extended time points. Therefore, we suggest that the observed changes in c-fos expression are more likely related to whether individuals encountered a remembered or non-remembered opponent, rather than reflecting a direct pharmacological effect of MK-801 on neuronal activity.

When considering all the nuclei together, we found that individual variability in neuronal activation can be clustered by treatment. In particular, our multivariate analysis suggests differences between individuals not exposed to physical interaction (NAE group) and those involved in social interactions, regardless of the treatment (MK-801 or Water). These results show the effect of physical interactions on the overall activity of the telencephalic nuclei. We also assessed functional connectivity among these brain areas by correlation analysis, co-activation patterns, strength analysis, and eigenvector centrality, since these are popular analyses to study the importance of brain areas from the SDMN in sociality in this species (10–13).

Nevertheless, functional connectivity analysis suggests differences in individuals exposed to physical interaction between treatments. Individuals exposed to a non-remembered opponent showed higher strength centrality and a trend toward higher connectivity among their nuclei and total connectivity than individuals exposed to remembered opponents. These results suggest that different telencephalic nuclei and neural networks may be differentially activated depending on whether fish are exposed to a known conspecific they do not remember, or to an already known and remembered opponent. Accordingly, we propose that social memory related to remembering an opponent, as well as differences between novel and familiar social stimuli, may be modulated not only by the activity of individual nuclei but also by the functional connectivity among them. This pattern suggests a more integrated neural network when individuals are exposed to a socially novel stimulus. It is important to note that all correlations were positive, suggesting co-activation across all nuclei. However, negative correlations were observed when evaluating other nuclei within the SDMN, as reported in other zebrafish studies (12).

Considering that the Dm nucleus is the suggested homology of the basolateral amygdala of mammals, which is involved in aggression, emotional learning, and memory (6, 25), and taking into account previous results in fish suggesting that this region is involved in these functions (58–60), we hypothesize that there could be differences in the activation of this nucleus when comparing individuals exposed to a recognized and remembered opponent with those exposed to an unrecognized opponent. However, we did not find these differences, nor did they appear to be a prominent nucleus in terms of connectivity with the other analyzed nuclei. It is important to emphasize that the cited literature does not evaluate individual recognition and memory in the context of social interaction and a previous agonistic encounter. Therefore, other regions may be involved or may exhibit greater relevance. Nevertheless, we do not rule out the possibility that assessing a greater number of nuclei belonging to the SDMN could reveal a more significant role for the Dm.

## Limitations

In this study, we present evidence of activation in four brain nuclei to compare neuronal activation between individuals who remember their opponent in a second encounter and those treated with the amnesic agent, with no memory of a previous encounter, thus facing a novel opponent. This interpretation is based on the previously mentioned behavioral data (29), as even when both experimental groups face the same opponents in two consecutive encounters, these opponents are not remembered when fish are exposed to the amnesic agent. However, an additional experimental group that would further strengthen our findings would be to assess the activation of these nuclei following a second encounter with a different opponent, allowing a comparison with the results obtained when administering MK-801 on the first day.

A potential confounding factor is that the interval between the first and second encounters varied between the groups: 24 h in the remembered opponent group and 48 h in the not remembered group. Nonetheless, both groups exhibited distinct behavioral outcomes during the second encounter, and neuronal activation was assessed immediately afterward. Based on the conceptual framework outlined above, these differences in neuronal activation are interpreted as reflecting either the reactivation of memory related to the previous encounter or its absence. Also, analyzing a larger number of brain nuclei and areas will allow us to generate a more enriched correlation network, enabling a stronger assessment of the role of each nucleus within that network. Additionally, it would allow us to evaluate the activation of nuclei unrelated to recognition and memory, serving as an internal control for the changes resulting from our treatments. Finally, while our network analysis shows all positive correlations and no negative correlations for any pair of nuclei, this may suggest functional co-activations and no co-inhibition between brain regions. In this sense, future studies should consider analyzing a broader set of genes and neural markers, as well as using double-immunolabeling techniques to identify which specific neuronal subtypes are involved in opponent recognition and retrieval of prior agonistic experience.

Another important factor to take into account is the potential influence of individual differences in personality. In recent years, numerous studies have shown that zebrafish can be categorized according to their behavioral responses to the same stimulus. For example, “bold” individuals may exhibit differences in social and aggressive behaviors, as well as activity levels, compared to “shy” individuals. These personality traits can also influence learning processes and the salience of stimuli (61, 62). Future studies should consider these intrinsic differences in order to better control inter-individual variability.

## Conclusion

Our results provide a first step in studying neuronal activation associated with the retrieval of a memory of a previous opponent and the effect of a social interaction with a non-remembered one. While we discuss our results in the context of the previously mentioned literature, it is important to note that, to our knowledge, there is no evidence in fish for neuronal activation related to the memory and recognition of an individual during a social encounter. Thus, we postulate that the Vv nucleus is associated with social interactions, that the Dl nucleus is relevant for individual recognition, and that analyzing connectivity between nuclei is important for fully evaluating the neural activity underlying social memory and recognition of a conspecific.

## Data Availability

The original contributions presented in the study are included in the article/Supplementary material, further inquiries can be directed to the corresponding authors.
