# Supplementary material for: Remembering the opponent: neuronal activation associated with social memory in zebrafish
Source: Front Vet Sci. 2026 Mar 20;13:1793342. doi: 10.3389/fvets.2026.1793342 (PMC13046556; doi:10.3389/fvets.2026.1793342)
Supplement: Supplementary file 1 [file Data_Sheet_1.DOCX]

**Supplementary material**


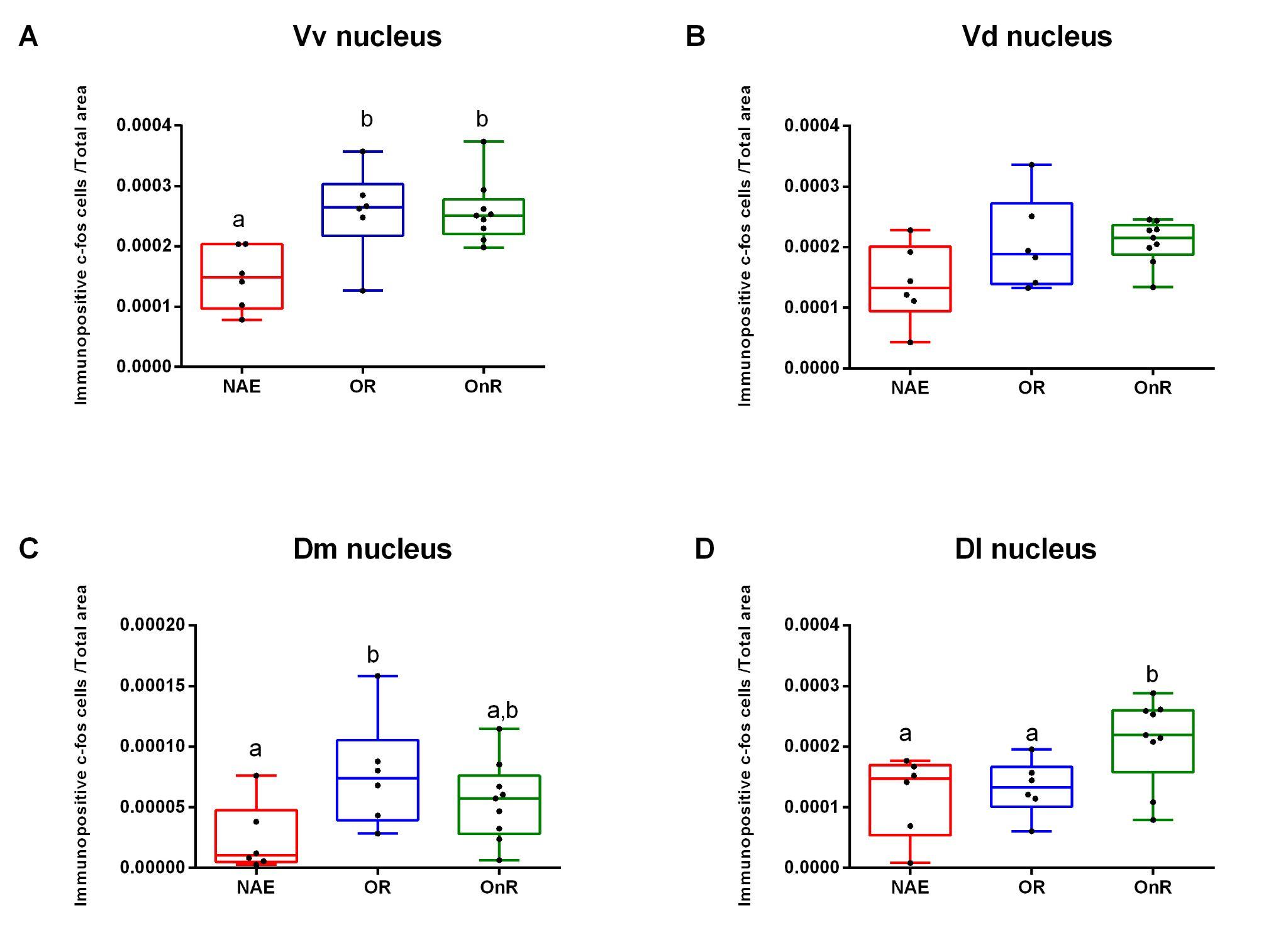


*Figure S1*

*Ventral Nucleus of the Ventral Telencephalic Area (Vv)*

When comparing the number of c-fos-immunopositive cells per total area among the three treatments (NAE, OR, and OnR), significant differences were found (Kruskal-Wallis chi-squared = 9.357, df = 2, p-value = 0.0092). Individuals not exposed to social encounters in NAE group show reduced immunopositive cells when compared to both groups of fish participating in social encounters (NAE: median: 0.000148 c-fos IC/TA, IQR: 0.000078, n=6; OR: median: 0.000264 c-fos IC/TA, IQR: 0.000028, n=6; p-value = 0.0060, Cohen's d=1.73), as well as in the OnR group (OnR: median: 0.000250 c-fos IC/TA, IQR: 0.000031, n=9; p-value = 0.0084, Hedges' g=2.15). No differences were found between the OR and OnR groups (p-value = 0.7085, Hedges' g=0.016, Fig. 2A).

*Dorsal Nucleus of the Ventral Telencephalic Area (Vd)*

When comparing the number of c-fos immunopositive cells per total area among the three treatments (NAE, OR and OnR), no significant differences were found (Kruskal-Wallis chi-squared = 4.6608, df = 2, p-value = 0.0972) (Median: NAE 0.000132 c-fos IC/TA, IQR: 0.000066; OR 0.000188 c-fos IC/TA, IQR: 0.000085; OnR 0.000215 c-fos IC/TA, IQR: 0.000030, Fig. 2B).

*Medial Nucleus of the Dorsal Telencephalic Area (Dm)*

When comparing the number of c-fos-immunopositive cells per total area among the three treatments (NAE, OR, and OnR), significant differences were found (Kruskal-Wallis chi-squared = 6.795, df = 2, p-value = 0.0334). Individuals not exposed to social encounters in the NAE group show reduced immunopositive cells when compared to fish participating in social encounters OR (NAE: median: 0.000012 c-fos IC/TA, IQR: 0.000025, n=6; OR: median: 0.000074 c-fos IC/TA, IQR: 0.000036, n=6; p-value = 0.0105, Cohen's d=1.48). Although no significant differences were found between NAE and OnR group, there is a trend with a p-value close to the defined significance threshold and a large effect size (OnR: median: 0.000057 c-fos IC/TA, IQR: 0.000023, n=9; p-value = 0.0691,Hedges' g=1.13). No differences were found between the OR and OnR groups. Nevertheless, a medium effect size was found (p-value = 0.3244, Hedges' g=0.64, Fig. 2C).

*Lateral Nucleus of the Dorsal Telencephalic Area (Dl)*

When comparing the number of c-fos immunopositive cells per total area among the three treatments (NAE, OR, and OnR), significant differences were found (Kruskal-Wallis chi-squared = 6.5541; df = 2, p-value = 0.0377). Individuals not exposed to social encounters in the NAE group show no differences in neural activation when compared to fish participating in social encounters OR (NAE: median: 0.000146 c-fos IC/TA, IQR: 0.000076, n=6; OR: median: 0.000132 c-fos IC/TA, IQR: 0.000037, n=6; p-value=0.9285, Cohen's d=0.23), they show reduced immunopositive cells when compared to the OnR group (OnR: median: 0.000219 c-fos IC/TA, IQR: 0.000045, n=9; p-value = 0.0284, Hedges' g=1.31). OR group showed reduced c-fos-immunopositive cells in Dl compared to the OnR (p-value = 0.0366,Hedges' g=1.25, Fig. 2D).

|  | Winner  (c-fos IC/TA) | Loser  (c-fos IC/TA) | P-value |
| --- | --- | --- | --- |
| Vv | Median: 0.000257  IQR: 0.000027 | Median: 0.000244  IQR: 0.000020 | 0.875 |
| Vd | Median: 0.000228  IQR: 0.000012 | Median: 0.000204  IQR: 0.000039 | 0.625 |
| Dm | Median: 0.0000588  IQR: 0.0000251 | Median: 0.0000467  IQR: 0.0000347 | 0.625 |
| Dl | Median: 0.000216  IQR: 0.000015 | Median: 0.000258  IQR: 0.00015 | 0.875 |

Table S1. c-fos IC/TA for winners (n=4) and losers (n=5) for Vv, Vd, Dm, and Dl nucleus (OnR). P-values for the Mann-Whitney test comparing winners and losers for each nucleus were shown.

A

| OnR | Vv | Vd | Dm | Dl |
| --- | --- | --- | --- | --- |
| Vv | 1 | 0.866 | 0.666 | 0.650 |
| Vd | 0.866 | 1 | 0.633 | 0.666 |
| Dm | 0.669 | 0.633 | 1 | 0.666 |
| Dl | 0.650 | 0.666 | 0.666 | 1 |

B

| OR | Vv | Vd | Dm | Dl |
| --- | --- | --- | --- | --- |
| Vv | 1 | 0.600 | 0.085 | 0.142 |
| Vd | 0.600 | 1 | 0.371 | 0.771 |
| Dm | 0.085 | 0.371 | 1 | 0.600 |
| Dl | 0.142 | 0.771 | 0.600 | 1 |

C

| Control | Vv | Vd | Dm | Dl |
| --- | --- | --- | --- | --- |
| Vv | 1 | 0.942 | 0.200 | 0.600 |
| Vd | 0.942 | 1 | 0.428 | 0.771 |
| Dm | 0.200 | 0.428 | 1 | 0.657 |
| Dl | 0.600 | 0.771 | 0.657 | 1 |

Table S2. Spearman correlation matrix for A) OnR group, B) OR group, and C) NAE. Spearman correlation coefficient was shown for each pair of nuclei.

|  | MWU | Permutation | Dunn's test |
| --- | --- | --- | --- |
| OnR vs. OR | 0.111 | 0.094 | 0.1572 |
| OnR vs. NAE | 0.573 | 0.516 | 0.4795 |
| OR vs NAE | 0.368 | 0.359 | 0.4790 |

Table S3. P-values of Mann-Whitney U, permutation, and Dunn`s test comparing total connectivity between each pair of groups.

A

| OnR | Strength | eigenvector |
| --- | --- | --- |
| Vv | 6.36 | 1 |
| Vd | 6.33 | 0.99 |
| Dl | 5.96 | 0.927 |
| Dm | 5.93 | 0.921 |

B

| OR | Strength | eigenvector |
| --- | --- | --- |
| Vd | 5.48 | 1 |
| Dl | 5.02 | 0.95 |
| Dm | 4.11 | 0.73 |
| Vv | 3.65 | 0.58 |

C

| NAE | Strength | eigenvector |
| --- | --- | --- |
| Vd | 6.28 | 1 |
| Dl | 6.05 | 0.94 |
| Vv | 5.48 | 0.88 |
| Dm | 4.57 | 0.66 |

Table S4. Strength centrality and eigenvector centrality for A) OnR group. B) OR group and c) NAE. The nuclei were ordered from highest to lowest for each treatment.
